# The US Food and Drug Administration Provides a Pathway for Licensing Vaccines for Global Diseases

**DOI:** 10.1371/journal.pmed.1000095

**Published:** 2009-07-21

**Authors:** Michael J. Brennan

**Affiliations:** Aeras Global TB Vaccine Foundation, Rockville, Maryland, United States of America

*An important breakthrough in US public health policy has the potential to accelerate the development of vaccines to prevent infectious disease epidemics that currently kill millions worldwide each year.*

Responding to an urgency for new vaccines for global diseases, in September 2008 the Center for Biologics Evaluation and Research (CBER) at the US Food and Drug Administration (FDA) published an important FDA Guidance Document (see Box 1), “General Principles for the Development of Vaccines to Protect Against Global Infectious Diseases,” [1] that should expand the FDA's role in facilitating the development of new vaccines for global bacterial, viral, and parasitic diseases affecting millions of people in developing countries worldwide. In part, this action is in response to a changing paradigm in advocacy for global diseases and recognition on the part of developed nations that the facilitation of vaccines and other products for neglected diseases disproportionately affecting those living in poverty is an effort that benefits industrialized as well as low-income countries. Moreover, through the effort of researchers, the pharmaceutical and biotechnology industries, and guided by nongovernmental product development partnerships, many funded by the Bill & Melinda Gates Foundation, preventative vaccines for global diseases are becoming a reality. Recent progress in this area is exemplified by both a meningococcal vaccine [2] and a new malaria vaccine moving forward into phase III efficacy trials in several African nations [3] and by several candidate vaccines for the prevention of tuberculosis being tested in phase I and II trials [4]. Vaccines and therapeutics for other viral [5], bacterial [6], and parasitic [7] neglected diseases are also in various stages of progress.

**Box 1. FDA Guidance Documents**

Guidance documents issued by the FDA contain nonbinding recommendations that represent the agency's current thinking on a specific topic. This is in contrast to regulations that appear in the US Code of Federal Regulations, which are legally binding and enforceable by the FDA. Approaches other than those discussed in the guidance document can be used by product developers if they satisfy the requirements of applicable regulations. Guidance documents are developed by individual Centers at the FDA according to designated responsibilities; for example, the guidance on vaccines for global diseases discussed in this report was developed by the Office of Vaccines Research and Review within CBER/FDA. The most common driving force behind the development of guidance documents is the identification of gaps or needs in manufacturing, product characterization, clinical review, and interpretation of policy that if addressed will facilitate the development of safe and effective products. Guidance documents are usually issued as draft documents open for public comment through the US Federal Register via http://www.regulations.gov/.

As these new products enter into clinical trials, it has become clear to product development partners that it is critically important to identify regulatory pathways leading to the timely evaluation and acceptance of safe and effective life-saving interventions. However, this effort is impeded by the fact that regulatory agencies in developing countries where tropical diseases are endemic often lack the capacity to review applications for new vaccines, resulting in lengthy delays in obtaining permission to conduct clinical trials. Better-resourced regulatory agencies such as the US FDA, Health Canada, and the European Union's European Medicines Agency (EMEA) have been willing to help strengthen regulatory authorities in developing countries, mostly through capacity-building and training programs coordinated by the World Health Organization (WHO), yet their ability to license new vaccines for global diseases has been restricted by the paucity of these diseases within their countries [8]. Some new approaches are being undertaken by the EMEA and by WHO and are briefly described here.

In 2004 the European Union introduced a resolution (Article 58) stating that the EMEA could provide a scientific opinion equivalent to a marketing authorization in cooperation with WHO for the evaluation of medicinal products intended exclusively for markets outside the EU community [9].

The intent of the statute was to provide sponsors of products for developing countries the opportunity to use the expert regulatory review services of the EMEA to evaluate the purity, safety, and effectiveness of a new product and to obtain a “certificate” equivalent to a European marketing license. In addition, Article 58 overcame an EU rule that required the withdrawal of an EU marketing authorization if the product was not marketed in Europe for three years. In order to be eligible for the Article 58 procedure, the product must be intended to prevent or treat diseases of major public health interest as defined by WHO. This list of target diseases includes major health threats such as HIV/AIDS, malaria, and tuberculosis as well as vaccines for possible use in the Expanded Programme on Immunization (http://www.wpro.who.int/sites/epi/) and to stockpile for emergency use. The dossier submitted by the sponsor for evaluation must be equivalent to that required for marketing authorization. A valid “scientific opinion” resulting from the review procedure can be used as a condition for prequalification by WHO for global distribution of the product (see below). Since the Article 58 process has been little used since its inauguration (the EMEA Web site indicates that three drugs for HIV/AIDS and one combination vaccine, Globorix, have been submitted for consideration; however, the vaccine was withdrawn by the sponsor prior to the opinion), questions remain such as: “What are the obligations of the manufacturer, WHO, and developing countries in the process?” and “Who is responsible for post-marketing surveillance and pharmacovigilance once the product is marketed in a country?”

The original intent of WHO's prequalification system established in 1989 was to assess the acceptability of vaccines for purchase by United Nations agencies for global distribution (see [10]). The goal was to provide the potential for more widespread global immunization with safe and effective vaccines to prevent the diseases that occur most frequently worldwide. The program relies on the ability of the national regulatory authority of the country of the product manufacturer to assure general safety and effectiveness of the product via its normal approval process. In addition, the national regulatory authority as well as the manufacturer are assessed (prequalified) using specific WHO criteria [11]. Also, distribution lots of vaccine are randomly check-tested according to WHO-specified criteria, surveillance methods are used to monitor immunized populations for adverse events, and the product often needs to successfully meet standards set by a specific WHO recommendations document. The “Prequalification” program is administered by the Immunization, Vaccines and Biologicals Department of WHO, and specific committees play parallel roles in assuring the quality of the product, such as the Expert Committee on Biological Standardization, which develops a specific recommendations document on the use of the vaccine, and the Global Advisory Committee on Vaccine Safety, which reviews and publishes reports on safety issues related to specific vaccines. Manufacturers apply for prequalification of their products to meet the needs of a list of priority products determined by UNICEF, the Pan American Health Organization, or recommendations from others such as the Global Alliance for Vaccines and Immunization. A licensed vaccine is normally required to implement the prequalification process, which can result in an added delay for introducing new vaccines; however, parallel and fast-track reviews can be requested for certain products for high-priority diseases, which can shorten the timelines of new much-needed vaccines. Licensure of a new vaccine for a global infectious disease via the FDA's new licensure process or through the EMEA's Scientific Opinion process can be linked to WHO's Prequalification program to ensure global distribution of the vaccine.

## “General Principles for the Development of Vaccines to Protect against Global Infectious Diseases”

To address the gap in regulatory pathways for global vaccines, the FDA's new guidance document provides an additional solution by indicating that (1) the FDA can license vaccines to protect against infectious diseases or conditions not endemic in the US, (2) the regulatory pathway is the same as for vaccines licensed for use in the US, and (3) the clinical data from trials conducted outside the US can be used for licensure. The principles in this document are supported by legislation, including the Food and Drug Administration Amendments Act of 2007 section 524, which recognizes the importance of accelerating the development of products that prevent diseases for which there is no market in the US. The document is an important declaration, for it ensures that a vaccine for a disease not endemic to the US can be considered for licensure if it has been shown to be safe and effective under the FDA's Investigational New Drug process. The Investigational New Drug process is particularly thorough in that it provides the vaccine sponsor with a complete review of (1) the purity of the product and (2) the nonclinical data, which ensures that the product is manufactured correctly each time. It also uses an independent analysis of the clinical trial results, which, along with the review by an FDA Advisory Board of experts, provides reliable advice on the safety and effectiveness of the new vaccine. Licensure by the FDA provides a strong foundation for countries that lack well-resourced regulatory agencies to consider this new vaccine for registration in their countries. As mentioned, it also facilitates WHO's ability to approve this new vaccine for global distribution through its vaccine prequalification and procurement process. The new FDA guidance also points out that vaccines for global diseases may meet the requirements for “accelerated approval,” which can significantly shorten the timelines for licensure in the US, or for designation as an “orphan drug,” which provides for a waiver of FDA user fees usually required for vaccine licensure.

To be precise, the FDA has, in the past, licensed vaccines for which there was limited disease in the US, in particular typhoid vaccine and Japanese encephalitis vaccine, although both of these vaccines were targeted in part for US military personnel and for US citizens traveling abroad [12],[13]. The new guidance reaffirms the FDA's ability to use foreign clinical data for licensure, as used in the examples above and also for the licensure of acellular pertussis vaccines for children in the US [14]. The recent licensure by the FDA of H5N1 influenza virus vaccine for the pandemic flu stockpile, for which no large efficacy trial was possible and little disease incidence exists, may also have helped provide a rationale for licensing a vaccine not endemic to the US.

## Issues, Challenges, and Implementation

It is clear, however, that the success of this promising FDA initiative will depend both upon the vaccine developers' willingness to submit their products to the rigorous FDA review process and on the FDA's ability to effectively implement the new recommendations. There are likely to be significant challenges for FDA reviewers. Although even a partially effective vaccine can have a major impact on diseases like tuberculosis, the FDA has traditionally not licensed vaccines with efficacy below 80%. The guidance document does not describe the principles FDA will use to license a vaccine that has efficacy levels unlikely to be accepted in the US but determined to be at a level that would save hundreds of thousands of lives annually in a country or a region where the disease is epidemic. Other questions are raised by the document. For example, how would a malaria vaccine for children be labeled for use if there is no US target population? Will developing countries be concerned that CBER/FDA could potentially use a different standard for review of products that will only rarely be used in the US? Will the cash-strapped FDA be given the resources to implement this new mandate? And finally, since this document addresses vaccines specifically, are similar licensing guidelines followed for drugs, therapeutics, and diagnostics?

Together with the new “priority review voucher” for tropical diseases, another program recently instituted by the US FDA to interest sponsors in developing products for tropical diseases (see Box 2), this new guidance offers paths for developers of vaccines for global diseases to access the regulatory experience and strengths of the US FDA. These new approaches are untested, and further public forums and continued surveillance as they are implemented should be encouraged to evaluate their contribution to global health. Nevertheless, the US FDA has given global vaccine developers some rules to play by, and the willingness of the FDA to engage in this global enterprise should be applauded.

**Box 2. Tropical Disease Priority Review Voucher**

Passage of the Food and Drug Administration Amendments Act of 2007 added section 524 to the Federal Food, Drug, and Cosmetic Act, which authorizes the FDA to award priority review vouchers to sponsors developing certain tropical disease products [15]. Products on the current list include: tuberculosis, malaria, blinding trachoma, Buruli ulcer, cholera, dengue, dracunculiasis, fascioliasis, trypanosomiasis, leishmaniasis, leprosy, lymphatic filariasis, onchocerciasis, schistosomiasis, helminthiasis, and yaws. Upon licensure of the product for the tropical disease, a voucher is issued to the sponsor that can be used to obtain a priority review for any vaccine or drug. Under “priority review status,” the FDA commits to the goal to review and approve (or provide a complete response letter to) an application no later than six months following receipt of the submission. This is advantageous for the manufacturer since it could translate into several months of market exclusivity and profit. In addition, vouchers can be transferred to another sponsor, which provides an opportunity for selling or trading the vouchers.

## Abbreviations

CBER: Center for Biologics Evaluation and Research
FDA: Food and Drug Administration
EMEA: European Medicines Agency
WHO: World Health Organization
